# Editorial: Advances in thyroid surgery and ablation therapy - treatment considerations in the era of quality of life

**DOI:** 10.3389/fendo.2025.1576425

**Published:** 2025-03-03

**Authors:** Tzu-Yen Huang, I-Cheng Lu, Gianlorenzo Dionigi

**Affiliations:** ^1^ Department of Otorhinolaryngology-Head and Neck Surgery, Kaohsiung Medical University Hospital, Kaohsiung Medical University, Kaohsiung, Taiwan; ^2^ Department of Otorhinolaryngology, School of Post-Baccalaureate Medicine and School of Medicine, College of Medicine, Kaohsiung Medical University, Kaohsiung, Taiwan; ^3^ Department of Otolaryngology-Head and Neck Surgery, Kaohsiung Medical University Gangshan Hospital, Kaohsiung Medical University, Kaohsiung, Taiwan; ^4^ Department of Anesthesiology, Kaohsiung Medical University Hospital, Kaohsiung Medical University, Kaohsiung, Taiwan; ^5^ Division of General Surgery, Endocrine Surgery Section, Istituto Auxologico Italiano IRCCS (Istituto di Ricovero e Cura a Carattere Scientifico), Milan, Italy; ^6^ Department of Pathophysiology and Transplantation, University of Milan, Milan, Italy

**Keywords:** thyroid surgery, thyroid ablation therapy, quality of life, patient safety, personalized treatment

In recent years, remarkable advances in thyroid surgery and ablation therapy have transformed the management of thyroid disease. These developments reflect a growing emphasis on precision, safety, and personalized care, as well as an increasing focus on optimizing patients’ quality of life (QoL). This Research Topic, “*Advances in Thyroid Surgery and Ablation Therapy - Treatment Considerations in the Era of Quality of Life*,” compiles 11 outstanding papers that address key aspects of this evolving field, including innovative surgical techniques, advanced technologies, and patient-centered treatment approaches.

A cornerstone of modern thyroid surgery is the ability to preserve critical anatomical structures while minimizing complications. Parathyroid gland preservation remains a major challenge, especially during reoperations for recurrent benign multinodular goiter. The study by Wang et al. highlights the use of carbon nanoparticles to enhance parathyroid gland identification and protection during reoperations, demonstrating a significant reduction in transient and permanent hypoparathyroidism. These findings underscore the potential of carbon nanoparticles to improve surgical outcomes in complex cases.

In addition to surgery, thyroid ablation treatments, such as radiofrequency ablation (RFA), are gaining traction as effective alternatives to traditional surgery for certain conditions. Kong et al. conducted a meta-analysis comparing RFA to surgery for papillary thyroid microcarcinoma and reported that RFA offers comparable oncologic outcomes with fewer complications, faster recovery, and better QoL. This shift toward minimally invasive options highlights the importance of patient-centered treatment paradigms.

Another significant advancement in thyroid surgery is the use of intraoperative neuromonitoring (IONM) to protect the recurrent laryngeal nerve (RLN) and external branch of the superior laryngeal nerve (EBSLN). Chiang et al. investigated the efficacy of transthyroid cartilage recording methods, emphasizing the superior electromyographic (EMG) signal quality obtained from contralateral electrode placements. Aygun et al. introduced cricothyroid muscle EMG to improve EBSLN monitoring, providing a complementary method to improve surgical precision and reduce the risk of nerve injury.

Technological innovations also play a pivotal role in refining surgical techniques and improving outcomes. Near-infrared autofluorescence (NIRAF) imaging, as described by Richard and Rizo, offers a promising solution for intraoperative parathyroid gland identification and viability assessment. The feasibility of combining autofluorescence and indocyanine green fluorescence angiography to assess parathyroid function is another notable development that bridges technology and patient safety.

Beyond intraoperative advances, preoperative and postoperative considerations are equally critical. The predictive models and nomograms developed by Liu et al. for lateral cervical lymph node metastasis in papillary thyroid carcinoma (PTC) exemplify the integration of diagnostic accuracy into clinical decision making. In addition, the findings in Zhang et al. provide insight into diagnostic accuracy for lateral cervical lymph node metastasis in PTC through nomograms, which facilitate personalized surgical planning. These tools facilitate personalized surgical planning, reducing unnecessary interventions and optimizing outcomes.

Pediatric thyroid surgery, a less common but highly specialized field, is addressed by Sosnowska-Sienkiewicz et al., who analyzed a decade of experience in the management of thyroid disease in children. Their findings emphasize the importance of multidisciplinary collaboration and surgeon expertise in achieving safe and effective outcomes for young patients.

Moreover, the study by Chen et al. on Delphian lymph node metastasis in PTC provides valuable insights into its prognostic implications and surgical management. This work contributes to a better understanding of central and lateral lymph node metastasis patterns, guiding individualized treatment strategies.

As the incidence of thyroid cancer continues to rise, the ability to predict and manage surgical risk remains paramount. Li et al. evaluated the utility of various EBSLN classification systems and concluded that Friedman’s classification provides superior predictive accuracy for EBSLN injury. These findings highlight the importance of adopting standardized classification systems in clinical practice.

Patient-centered outcomes such as voice and swallowing function are increasingly recognized as essential components of QoL. Zhao et al. examined subjective and objective analyses of these functions, providing critical data to inform treatment planning and postoperative care. The findings reinforce the need for a multidisciplinary approach to optimize functional outcomes.

This Research Topic highlights the significance of integrating advanced technologies, precision diagnostics, and patient-centered care into thyroid surgery and ablation therapy. The collective insights from these articles pave the way for safer, more effective, and QoL-focused thyroid care. We hope this compilation inspires continued innovation and collaboration among thyroid specialists, ultimately benefiting patients worldwide.

